# That eagle covering me: transitioning and connected autonomy for emerging adults with cystinosis

**DOI:** 10.1007/s00467-014-2921-5

**Published:** 2014-08-27

**Authors:** Maya Doyle, Allison Werner-Lin

**Affiliations:** 1 Division of Pediatric Nephrology, Children’s Hospital at Montefiore, Bronx, NY USA; 2 Department of Social Work, Quinnipiac University, Hamden, CT USA; 3 University of Pennsylvania School of Social Policy and Practice, Philadelphia, PA USA

**Keywords:** Cystinosis, Transitioning, Adherence, Emerging adulthood, Grounded theory

## Abstract

**Background:**

Rare diseases pose transitioning challenges owing to limited provider expertise and changing healthcare systems. The timeframe and developmental changes of emerging adulthood overlap with the transition of patients with cystinosis from pediatric to adult-oriented healthcare.

**Methods:**

This study utilized techniques of qualitative grounded theory to explore the experiences of adults aged 18–47 with cystinosis, and their parents, with a focus on the transition to adulthood and adult-oriented care. Forty-six individuals from 21 families were recruited online and at cystinosis conferences to participate in focus groups and/or individual interviews. The constant comparative method was used to conduct both line-by-line and focused coding of verbatim transcripts.

**Results:**

The following elements were reported to be critical to the transition to adulthood and adult-oriented care: gaining skills and responsibility for disease management, progressing toward autonomy while remaining connected to caregivers, and having strong communication with and between providers.

**Conclusions:**

Data analysis identified behaviors and relationships that support and/or threaten autonomy and treatment adherence. Participants described institutional, relational, and practical barriers to transition. Suggestions for improving transitioning include: identifying patient/family strengths and improving pediatric–adult provider partnerships and communication. Further research is needed into the experience of patients before and after transition to adult-oriented care.

## Introduction

Nephropathic cystinosis is a rare autosomal-recessive metabolic disorder with an estimated prevalence of 1 case per 100,000 to 200,000 live births [1]. There are believed to be 500 nephropathic cystinosis patients in the USA and 2,000 worldwide, with about 15 new diagnoses per year [1]. Individuals diagnosed in infancy and early childhood face Fanconi syndrome, growth delays, kidney failure, impaired vision, and numerous other systemic health challenges [2, 3]. Treatment involves a demanding medication regimen and eventual kidney transplant. Before the availability of cystine-depleting treatment, children with cystinosis reached end-stage renal disease at age 9 or 10 [1, 3], requiring kidney transplant, and faced early mortality by adolescence.

The availability of treatment in developed nations has transformed cystinosis from a fatal to a chronic disease [4, 5], with the median age of survival currently at 27–30 years [6]. Patients in the USA and other developed countries are now surviving into their 5th decade of life and beyond [3, 7]. The need for kidney transplant is now commonly pushed back to late adolescence and emerging adulthood [1, 6, 8]. Greater availability of the oral medication Cystagon™, following Food and Drug Agency (FDA) approval in 1994, allowed for the slowing of disease progression [9] and for survival into adulthood with lower rates of complications and death in the USA and Europe [2, 10, 11]. Adherence to the treatment regimen has been correlated with improved outcomes [7], but the side-effects of Cystagon™, particularly gastro-intestinal distress [12, 13] (including nausea, vomiting, diarrhea), and a distasteful odor on the skin and breath, have created challenges to adequate adherence in early adulthood [4, 6]. In 2013, the FDA approved an extended release drug, Procysbi™, which promises to reduce side-effects and simplify patients’ medical regimens [13–15] via 12-h dosing. Also approved by the FDA in 2013, Cystaran™ eye drops [16] replaced the cysteamine eye drops previously only available on a compassionate basis; the regimen continues to include eye drops used once every waking hour.

### Transitioning to adult systems of care

The term *transitioning* in the medical context first came into use in the late 1980s, inspired by Surgeon General C. Everett Koop [17], who observed that neonates whose lives he had saved through surgery were now surviving into childhood and adolescence (not unlike the survival of children with cystinosis in the wake of cysteamine). A 2002 consensus statement standardized the definition of healthcare transitioning from pediatric to adult care as a process to “maximize lifelong functioning and potential through the provision of high-quality, developmentally appropriate healthcare services that continue uninterrupted as the individual moves from adolescence to adulthood” [18].

The age range 18–25 (or even up to age 30) has been identified as a time of rapid but normative developmental change, as individuals move beyond childhood, while postponing the fully fledged responsibilities of adulthood [19]. Arnett characterizes “emerging adulthood” by five main features: identity exploration; a sense of instability; a focus on self; a feeling of being “in-between”; and a sense of possibility [19]. For individuals living with cystinosis, the transition of care from pediatric to adult-oriented care overlaps with the timeframe of emerging adulthood, expectations for a greater level of autonomy in disease and treatment management, and changing educational, work, and insurance configurations.

The cystinosis community has identified the transition to adulthood and adult-oriented care as a priority. Services in resource-saturated pediatric systems of care often end abruptly as patients move to adult-oriented providers. Patients and providers may need time to connect personally, and across systems or cultures of care [20]. Adult-oriented health providers may be less prepared to take on the care of former pediatric patients, particular those with rare diseases [21, 22] and may respond with trepidation or reluctance [23, 24], particularly when conditions are evolving or new to their practices. Community efforts to highlight the needs of newly adult patients include the creation of a transition guide for patients and families [25], attention to patient- and family-oriented needs at professional and consumer conferences, and advocacy-group presence at meetings of adult-oriented nephrologists.

The ability to treat the condition does not equal cure, and the long-term sequelae of survival with cystinosis, its treatments, and the challenges of adherence remain to be understood. This qualitative grounded theory study included focus groups and interviews (Table 1) with adult cystinosis patients and their parents, exploring experiences of living with cystinosis in the context of changing medicine and the movement from pediatric to adult-oriented systems of care.

**Table 1:** Study participants by age group (emerging adults 18–30 years of age with cystinosis; adults 30 + years of age with cystinosis; parents of adults with cystinosis), gender, and data collection type (focus group, individual interview, or both)

Participants by age, gender, and data collection type	Participated in focus group only	Participated in individual interview only	Participated in individual interview *and* focus group	Total participants
Men aged 18–30	6		2	8
Men aged 30+	4			4
Women aged 18–30	1	4	3	8
Women aged 30+	2			2
Mothers	8	3	2	13
Fathers	8	2	1	11
Total participants	29	9	8	46 individuals (from 22 families)

## Materials and methods

### Sampling and recruitment

New York University‘s Committee on Activities Involving Human Subjects (UCAIHS) approved the study protocol, which originated in the School of Social Work. Inclusion criteria required participants to be: either 18 years of age or older with a diagnosis of cystinosis, or a parent of an individual 18 years or older with a diagnosis of cystinosis. Participants were recruited online through cystinosis advocacy organizations and in person at three cystinosis conferences. Recruitment for, or participation in, research studies during such conferences is not uncommon, and the community has demonstrated a high response rate to recruitment in other studies [26, 27]. Interested parties completed a brief online or paper questionnaire to provide contact information confidentially and to screen for inclusion/exclusion criteria. All participants received information about the study and gave informed consent before participation. Additional recruitment continued via purposive and nominations sampling.

### Data collection

**Fig. 1:**
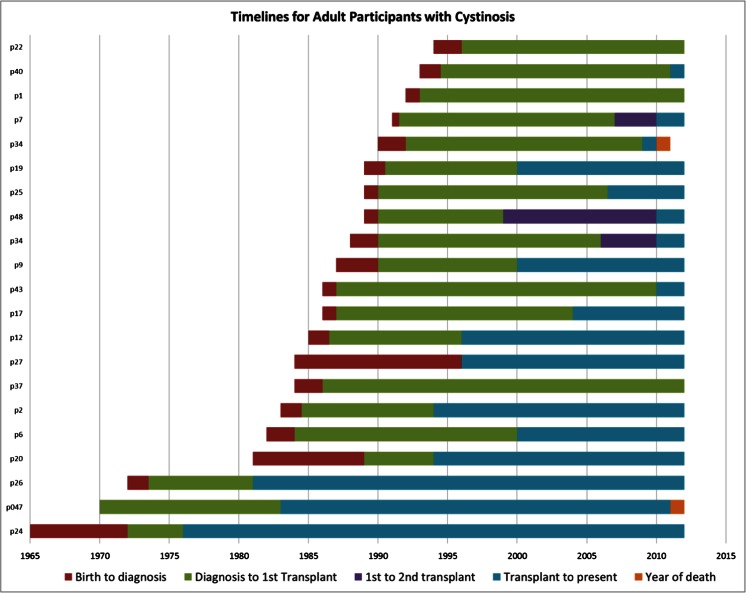
Timelines for adult participants with cystinosis: time to diagnosis, first and second transplant, transplant to present, and death, as of 2012. Note: after taking part in two focus groups, participant P047 died in 2012, after qualitative data collection had ceased. The parents of P34 contributed information, participating in a focus group and family interview, the year following the participant’s death.

Interview guides for both focus groups and interviews were developed specifically for this study (Table 2). Each participating adult with cystinosis completed a demographic questionnaire (age, gender, education, employment and income) and health history (age at diagnosis, transplant status, and age at transplant; Fig. 1). Six focus groups were conducted between July 2011 and April 2012 at the Cystinosis Research Network’s bi-annual Family Conference, the Midwest Cystinosis Families gathering, and the Cystinosis Research Foundation’s annual Day of Hope meeting. These conferences bring together cystinosis patients of all ages, family members, healthcare providers, and researchers from across the USA and around the world. Content typically includes forums focused on disease education, current treatments and those under investigation, disease research updates, psychosocial issues for patients and families, and opportunities for socialization.

**Table 2:** Interview guide for adult and parent participants

Interview guide	Adults with cystinosis (age 18+)	Parents of adults with cystinosis
Transmutation of disease/illness	How has having cystinosis changed as you’ve gotten older? What changes in the treatment of cystinosis have impacted you?	How has having cystinosis changed as your child has gotten older? What changes in the treatment of cystinosis have impacted them?
Emerging adulthood	Do you think of yourself as an adult? How do you define adulthood?	Do you think of your child with cystinosis as an adult? How do you define adulthood?
Transitioning	What are/were your concerns moving from pediatric to adult care?	What are/were your concerns moving from pediatric to adult care?

Seventeen individual semi-structured interviews were completed between July 2011 and June 2012 (Table 1), with adults with cystinosis and separately with parents who responded to recruitment efforts, some of whom also participated in one or more focus groups. Interviews generally took place in participants’ homes and lasted 90–120 min.

Focus groups and interviews were audio-recorded and professionally transcribed. In the tradition of grounded theory, field notes were written immediately after each focus group or interview. Also, a case history was written for each patient/family. Data collection continued until theoretical saturation (the point at which qualitative data collection and analysis yield additional language and incidents from participants, but generates no new codes or categories) [28] was reached.

### Data analysis

Substantive coding of transcripts was carried out in the grounded theory tradition of constant comparative analysis, in which data collection and analysis are simultaneous and iterative [28–30]. This methodology was selected because of its frequent use in exploratory, qualitative health research, and its focus on conceptualization over description. Atlas.ti 7 (qualitative data analysis software) was used to organize transcripts, codes, and memos [31].

Data analysis began with line-by-line coding of the first three focus group transcripts to identify a preliminary list of codes. These included open and in vivo codes (participant’s own words), as well as focused codes addressing features of emerging adulthood [19] and healthcare transitioning. For the most part, the analysis utilized gerunds (−ing words) in coding [32]—actions taken by participants to address their main concern(s)—rather than descriptions of psychosocial status. Focused coding was also utilized to identify specific psychosocial and medical details, such as education, work, relationship status, childbearing status, medical progression, medical regimen; this coding schema was useful in mapping participants’ illness and developmental trajectories. A codebook was created listing code definitions and parameters, brief memos, and examples of each code, followed by another round of coding to collapse and clarify emerging codes. To facilitate translation of codes into findings, codes were grouped into: individual, family, and community contexts; medical issues; healthcare transitioning; and emerging adulthood.

When necessary in reporting findings, participants are described by age, gender, or parental role, but demographic information is used as little as possible (see the section Strengths/limitations). Phrases in *italics* denote codes that emerged through constant comparative analysis. Quotes are included to further illustrate codes and share the experiences of cystinosis patients and families with readers.

## Results

### The recognition of reprieve

“Wouldn’t it be cool to grow old?” declared one emerging adult participant.

Participants were cognizant that, because of changing medicine, both cystinosis patients and their families experienced a *reprieve* from the previously fatal nature of cystinosis. Individuals and families *recognize the reprieve* over time and confront its variable impact on their health and psychosocial status. Participants described how they had *outlived the prognostication* of an early demise given to their parents at the time of their diagnosis. Figure 1 illustrates the diagnosis and transplant trajectories of participants, particularly showing an increase in time to transplant. Participants recognized how medical advances changed their expected lifespan and quality of life. In stable if not perfect health, many expected to live into adulthood, complete schooling, work, form romantic partnerships, and become parents.

Parent participants spoke of celebrating birthdays that they were told their child would not live to see: “The prognosis they gave us was she probably won’t live to see her 16th birthday. When it came time, I started planning a party…you’re here, and we’re going to have a party.”

### Transitioning

*Outliving prognostication* was accompanied by the need to transition from pediatric to adult-oriented care. This shift brought both opportunity for independence, and much fear and anxiety on behalf of cystinosis patients and families. Participant mothers shared how frightened they were at the prospect of losing trusted relationships with healthcare providers, and a level of attention and communication that had helped keep their children alive:

It’s good now. It was horrific for a year, the transition period, when he was leaving the pediatric doctor and going to the adult…I cried—the pediatric people we’d gotten so attached to. I mean you call them on the phone and you get a call back within 20 minutes, which hasn’t happened at all in the adult world. The pediatric people knew cystinosis better than anybody, and had managed to get him from near death to where he is today.

Participants shared how transitioning was not just a time with a “sense” of instability [19], but a time of real risk. They experienced health- and life-threatening lapses in treatment because of not-uncommon challenges to medication adherence, the change in care paradigms, and the lack of adult-oriented providers who are knowledgeable about this rare disease.

When I was in college I actually went four years without going to a doctor at all, but I would always call the doctor for my medicine.

His doctor, knowing he’s going off to college, just had a come-to-Jesus talk with him and said, I’ve got a kid right down the hall from me whose creatinine was 1 and now it’s around 6. He said, I don’t want you to be one of those statistics where this age group is very much at risk.

There was a big percentage of patients with cystinosis who’d had transplants as children, and within two years of moving into adults they lost the kidney

We started talking about [transition] when I was 16. It was a bit difficult because we couldn’t go anywhere that knew anything about cystinosis. We went [to another hospital] for one consultation…They basically came out with this bottle and said, oh, can you do a 24-hour urine count? So I thought they obviously don’t know anything about cystinosis if they don’t know how much we [urinate].

Communication between providers was a particular source of *instability* within the transition process. Participants and families expressed concern about the gaps *between* pediatric and adult providers.

There’s not as much communication as you would think between the pediatric area and the adult nephrology area. They pass their charts along, but there’s not [communication]. Maybe that’s something that needs to happen, more of a transition between the doctor’s departments so that when these kids are moved on, there’s communication.

So it was letting him, number one, be in charge of everything, and then trusting that they’re going to be able to understand this disease well enough to keep him as healthy as he’s been.

There’s no central connecting point. I go to this doctor, I go to this doctor, I go to this doctor, and they all fill out the files that go into the same pile, but as far as direct communication between them, I don’t think there’s much.

If they could develop some sort of a serious transition area between pediatric and adult with communications between—so many kids did great in this [renal] population and are dead within five years of this move.

Participants praised institutions that have created transitioning clinics or otherwise facilitated the transitioning process to provide continuity and stability:

When I went to the adult side of things, we just got stuck into renal, whereas, cystinosis can affect all different areas. So, you don’t get seen for the other bits. So [our adult nephrologist] set up a cystinosis clinic, so everyone can see him for the renal, but he’ll do it so on the same day they can get [seen] for other things, ophthalmology, EKGs, speech and language therapy, things like that.

### Regimenting

Participants reported the importance of *regimenting*, creating systems and structures to organize the management of cystinosis. Over time, *regimenting* by parents sets the stage for the *transfer of power* to greater self-management in adolescence and adulthood. One participant, still in high school, described, “catering to the medications,” adjusting her school schedule and activities around side-effects, particularly fatigue and stomach upset. Participants described the use of spreadsheets, care binders, watches or phones set with reminder alarms, pill boxes organized by day and time that track whether medications have been taken, or a “go-bag” ready for outpatient visits and hospitalizations. Such tools supported adherence and increasing autonomy, vital components of the transition to adult-oriented care.

The pharmacokinetics of cysteamine and other supplements, and the risk of rejection of a transplanted kidney, make dose timing critical [33]. Parents described providing age-appropriate but straightforward information to their children about why the medications were so vital, recognizing (or at least hoping for) the reprieve that treatment offers and trying to make consequences clear (whether disease progression or increasing medication doses). Emerging adult participants recalled when, during childhood, their parents enforced the idea that medication-taking and medication time were not flexible, but rather a usual part of one’s day, “like brushing my teeth…an everyday thing that I had to do.” Participants described how family strategies like these *acclimated* them to their medication regimen, even though adherence remained at times challenging, particularly during adolescence and the move to college and/or independent living.

The medical regimen to manage cystinosis is more complex than the tooth-brushing analogy implies, as a participating parent clarified, “54 pills and over 800 milliliters of liquid medication every 24 hours”. Adult participants and their parents described the challenge of fitting their complex regimen into their day, particularly during college and work:

I don’t think there’s anybody that takes as much medicine as he does that doesn’t forget once in a while.

The eye drops are the hardest to remember because they’re every hour. You would think it wouldn’t be because they’re every hour. It’s the total opposite, especially when you’re sitting in a three-hour class.

It gets in the way. Sometimes it’s as easy as you’re so busy at work, you skip it once, and then it becomes twice.

It’s almost impossible for me to take the middle of the night. I mean, I try to always get 3 but I never really try to get the last one.

While *regimenting* provides skills and habits to support adherence, participants reported that therapy-related factors [34] (particularly the odor and side effects of cysteamine) dictated many adherence-related decisions, particularly as participants entered into social and romantic relationships as adolescents and emerging adults. Participants also reported systemic and socio-economic factors [34], particularly insurance coverage and drug costs, which support or deter adherence. Echoing the view of adults with cystinosis that financial stability was a part of their transition to adulthood, parents described fears that, despite their efforts at regimenting, financial instability might result in hidden non-adherence, “because they don’t have any money and they don’t take their medicine.”

### Transferring power

As children with cystinosis enter adolescence and then adulthood, families begin *transferring power* over disease and treatment management from parent to child. This developmentally appropriate task is made more challenging by the need to manage the disease, while also experiencing other transitions (in healthcare systems, living situations, education, etc.). Both adult and parent participants discussed reluctance or ambivalence about this transfer; parents were reluctant to allow an adolescent or emerging adult to take greater responsibility in their care, whereas some emerging adults were ambivalent about accepting it. One participant felt that he needed to “step forward” and take over his care as a way of confronting his parents’ fears and making them “more comfortable” with his self-management.

Adult participants and parents expressed anxiety around what could (or did) go wrong during attempts at *transferring power* from parent to child. Parent participants feared that their diminished participation in their child’s care could decrease adherence and result in disease progression or death. They struggled to relinquish control: “We’ve been so focused on keeping them healthy and keeping up with their meds. It’s hard to give that control over to them.”

Emerging adult participants approached the *transfer of power* with trepidation as well. One admired how his mother had managed all of the information and tasks involved in his care, and feared his inability to do those things as well as she had:“I never can remember as much as she does because she worried so much, she always had it pounded in her brain and had it organized.”

Parents and emerging adult participants defined self-care similarly, as a debt repaid, a fair trade for the years parents have worked to protect their child’s health:

We’ve done the right things. We’ve gave your medicine, we’ve made sure you’ve taken it. We’ve got you to school; we’ve got you to your appointments. It’s time that you take over part of this. You need to do this.

Our parents having control of it all of our lives and then us moving out, it’s hard on them. They feel very overprotective because of the issues you have. It’s tough on me too because they did so much for me.

### Defining adulthood

The process of transitioning from pediatric to adult-oriented care imposes a medico-legal definition of adulthood to which participants had to respond (at age 16, 18, or 21, depending on the medical center), whether they personally defined themselves as adults or not. One participant’s first hospitalization in an adult medical unit forced a recognition, with some shock and sadness, that “Oh my gosh, I’m an adult…I finally get a room and they bring me up to the room and they tell me that my mom can’t stay. I remember just lying in the bed and watching my mom walk down the hallway.”

The criteria participants used to define adulthood were often pragmatic, including responsibility for specific tasks and awareness of consequences of lapsed care. Participants did not rely on traditional demographic markers, such as finishing education, marriage or achieving parenthood [35]. When asked to define adulthood, participants with cystinosis tied definitions of adulthood to self-care and financial self-support: “You’re on your own, making your own decisions, living with the consequences of those decisions, and being able to financially take care of yourself.”

Participants described thinking of oneself as an adult as a process, not a specific moment in time. They saw themselves as “in between,” “not there yet,” or as an “incomplete adult.”

I have to understand my disease and have the doctors and stuff squared away. I know I can do everything; it’s just the getting there.

I feel like I’m in between. I don’t quite feel like a high school kid, but I don’t quite fee like a complete adult. I’m an incomplete adult.

I feel like an adult if I’m paying my own bills and all that stuff, not needing their help for that. That’s what I find difficult, being a complete adult.

Parent participants also tied perceptions of adulthood to self-care and financial management. Some noted concerns about maturity and recognized that their perceptions of their adult children with cystinosis were changing (or needed to).

I still have trouble thinking of [him] as an adult because he still lives at home with us. And there are things that he still just doesn’t do.

I’m hopeful he’ll be able to get a job, be independent, take care of himself. That would be icing on the cake of his life, being able to do those things.

[She’s] so organized; she takes care of everything. But she’s still my baby/

I feel like we’re just starting to think of him as an adult.

### Connected autonomy

Adult participants with cystinosis described wanting a sense of *connected autonomy*, being independent yet staying connected to their family. They were willing to ask for and accept help, both in daily living and in the event of a medical crisis. In maintaining *connected autonomy*, emerging adults (and adults) steered the management of their illness and their healthcare, and involved family and significant others as necessary. They reported the ability to call on family to help instrumentally and emotionally when needed. Depending on the participant’s health status and age, such help entailed accompanying patients during medical visits, helping with prescription refills, talking with college staff, managing childcare or domestic tasks, or giving input into healthcare decisions.

When I was in the hospital, after transplant, I needed some blood. I was like, ‘oh, I don’t want it.’ But my mom was there and said ‘you need it.’ She helped me make the right decision. It was my decision. I had to sign the consent. A lot of those medical choices I made myself but with the help and opinion of my mom or family.

One mother spoke practically about planning for the challenges of her daughter living independently (given her muscle weakness), envisioning a future in which she would be involved in her daughter’s daily life:

If she was going to be living on her own, things that I do for her, how can we make it that she can do things for herself? If she were going to be doing her own grocery shopping, she can’t buy a gallon of milk. She would have to buy a half a gallon. So that she can carry it and tip it. Independence is going to take a lot of planning…a lot of assistance.

For some participants, the need to rely on family was a source of disappointment, and a threat to autonomy:

I had some health issues so I moved back home, which was very difficult for me because I like to be very independent, and the whole reason I think I moved [away] was because it was basically the farthest that I could go.

For others, *connected autonomy* provided the safety of knowing that parents would be there when needed, even while adults with cystinosis were striving to live independently.

They’ll always be there to help me, but I just want to let them know I can hang out myself and if I ever need any help I know they’ll be there.

You always want help. Help’s great. But at the same time you want them to carry on with their lives too.

For partnered young adults, *connected autonomy* extended from family of origin to family of choice. Adults with cystinosis who entered into long-term relationships considered the implications of chronic illness and possible progression, the need for assistance, now or in the future, and decisions about child-bearing or adoption. Yet, they reported expectations and motivations of partners and parents that were quite different. One participant described how her mother “screened” her fiancé, recognizing a need for a partner willing to be a caregiver. When that relationship was unsuccessful, this participant envisioned her parents as protective eagles watching over her:

I do feel that I need to be with my parents because of cystinosis. I’m not just transplanted. I have cystinosis. I do need that protection still. I don’t think my kidney would have failed if I was with them. Because my mom—she wasn’t begging me, but she would say, did you take the medicine? So I feel like I do need that protection, *that eagle covering me.*

## Discussion

The movement from *regimenting* by parents, to *the transfer of power* throughout adolescence and emerging adulthood, the roles and responsibilities seen as part of *defining adulthood*, and the maintenance of *connecting autonomy* represents a positive pathway for the management of illness and improved outcomes for those diagnosed with cystinosis in childhood. Reflective of Arnett’s features of emerging adulthood [20], participants described *being in-between* in their definitions of adulthood and in descriptions of institutional and practical barriers to transition from pediatric to adult-oriented care, and shared concerns about *instability*, personally, financially, and medically. They also spoke of their lives with a great deal of positivity and *possibility*, as they pursued higher education, traveled, formed long-term relationships, and started families.

The *reprieve* offered by changing medicine over the last few decades does not guarantee a smooth or positive trajectory throughout the lifespan. As Fig. 1 details, the age at transplant for many participants is now in late, rather than early, adolescence—sometimes occurring just as they are starting college or living with peers. Aspects of the disease may force a step back from developmentally appropriate autonomy to greater dependence—be it a medical crisis, a need for dialysis, transplant, or transplant rejection. Such episodes may reverse the *transfer of power*, at least temporarily. Health concerns, combined with other socio-economic drivers, may maintain or return emerging adults to the nest. At times a disappointing realization, it is difficult to let go of autonomy once one has claimed it. Providers may consider tempering expectations of self-management and autonomy as part of transitioning with a realistic need for support throughout the lifespan, and a developmentally appropriate renegotiation of roles.

### Transitioning

Adult patients with cystinosis and parents both identified how these adults are attempting to assume greater responsibility for self-care with fewer institutional supports and in a new and unknown medical environment. Ongoing efforts within the professional pediatric nephrology community seek to address gaps in transitioning for the broad spectrum of pediatric renal patients [36–39]. The cystinosis community has identified healthcare transitioning and the transition to adulthood as priorities for research and programming [25]. Results from this study and from others [40] suggest that transitioning must be viewed as a process, a flexible one at that, not simply a transfer date (one to be approached with dread). Within pediatric patient–family–provider relationships, building self-care and self-advocacy skills before the transfer of care seems paramount. These skills many benefit both adherence and interactions with future providers. Simultaneously, education and engagement of adult-oriented nephrology teams (and other specialists and primary care providers) in assuming the care of patients now surviving with cystinosis is vital. The visible demonstration of communication and partnering between pediatric and adult-oriented providers can give assurance to transitioning patients and families that they are gaining new allies, not losing a trusted safety net. To support such relationship-building, cystinosis advocacy groups have taken opportunities to exhibit at professional conferences for adult-oriented nephrologists, such as the American Society of Nephrology’s Kidney Week.

### Adherence and practice implications

As described by participants, the survival of individuals with cystinosis relies in no small part on the effort, time, dedication, and focus of families in both obtaining and maintaining adequate treatment and fitting illness into family life. There is no *reprieve* without treatment. The challenges of medication adherence for adolescents with renal disease have been well-documented [36, 41, 42]. The challenges of adherence with cystine-depleting agents are recognized by researchers and healthcare providers, and continue to have an impact on the search for more tolerable and longer acting treatment options. Despite the promise of research in the areas of stem cell transplant and gene therapy [43, 44] and recent FDA and EMA approval of two drugs for cystinosis [13, 16], patients and families face a heavy task burden in managing the illness. Recognizing patient and family strengths, building on the *regimenting* styles and tools they have adopted, and communicating this understanding to accepting adult-oriented providers, may make transitioning a smoother, more positive process.

Inability to afford medications has been a major concern for adults with cystinosis, and their families, in the USA. Insurance coverage and healthcare utilization has been an area of particular instability for emerging adults [45, 46]. For those with cystinosis, changing coverage and access to care may create real risk. Families responding to the 2011 Living with Cystinosis survey [27], a precursor to this study, expressed great concerns in this area. While most families (in the USA) were relieved by the extension of dependent coverage to age 26 and the elimination of pre-existing condition criteria as a result of the Affordable Care Act [47], some were fearful of losing access to life- and transplant- preserving medications if they, or their families, lacked insurance coverage or adequate finances. It is imperative that transitioning discussions with cystinosis patients and their families include plans and resources for maintaining health coverage and financial stability [40].

### Strengths/limitations

Criteria for assessing qualitative and grounded theory research include trustworthiness [48], credibility, usefulness [29], transferability, and confirmability [30]. Interview guides for this study were developed with key informants, including adult and adolescent members of the cystinosis community, parents, healthcare providers, and other researchers. Peer debriefing [30, 49] was available from fellow social work and health researcher’s participation in the Cystinosis Research Network’s Adult Care Excellence initiative, in developing the Living with Cystinosis survey [27] and the Bridges to the Future Transitioning Guide [25] also provided for prolonged engagement [30, 49] and insight into the concerns of the community. Themes similar to the concepts identified in this study have also been described by patients and families in advocacy organization newsletters and social media, providing triangulation [30, 49] with publicly available information.

The specific disease population, sample size, qualitative nature, and methods of this study provided rich data regarding the experiences of adults with cystinosis, which may or may not be comparable with the experience of individuals with other chronic and rare diseases, or with other birth cohorts affected by cystinosis. The sample is based primarily in the USA; the experience of living with cystinosis, and of transitioning to adulthood and adult-oriented care, outside of the USA and/or other developed nations, with potentially limited access to cystine-depleting therapy, and differences in organ allocation for transplantation, may be quite different [50, 51].

One risk of the “thick description” made possible through this qualitative method is the risk of inferred identification [52]; the small size and high level of connectedness within the cystinosis community increases challenges to anonymity and confidentiality. This presents a particular challenge in the dissemination of findings with meaningful detail, while protecting the identity of individual participants who are well known to each other, to providers, and to other researchers. Classic grounded theory methodology [53] helped to organize data around codes and concepts rather than around descriptions of individual participant or family experiences.

## Conclusions

Findings from this study conceptualize behaviors and relationships that support and/or threaten autonomy, disease self-management, and treatment adherence during this time of transition to adulthood and adult-oriented care. Imbuing individuals diagnosed in childhood with cystinosis, or other forms of rare or chronic illness, with the skills and philosophy for self-care, negotiating the *transfer of power*, and creating a sense of *connected autonomy* allow them, as adults, to engage in age-appropriate developmental tasks. The progressive nature of the illness, and the demands of medication regimens, underlie much (but not all) decision-making around higher education, vocations, romantic relationships, and family planning. Further research into the experience of emerging adulthood and adulthood with rare and chronic disease, during and following transition to adult-oriented care, is needed to continue improvements in quality of care, health outcomes, and quality of life.
